# Drying Cavities Preparatory to Filling

**Published:** 1855-10

**Authors:** 


### DRYING CAVITIES PREPARATORY TO FILLING.

All are aware of the importance of having the cavity and the gold perfectly dry, in filling teeth. Various expedients are resorted to to get rid of the moisture. Cotton wool, prepared flax fibre, tissue paper, buckskin, &c., have all been used, and with any of them, the moisture is mostly removed, but absolute dryness is not secured. This may be obtained by throwing a jet of warm air into the cavity, after wiping out with cotton. A convenient, and perfectly successful mode of effecting this is to construct a small blow-pipe, with a chamber, after the general form of Berzelius' blow-pipe. This chamber may be a cylinder $\frac{1}{3}$ or $\frac{1}{2}$ inch in diameter, and $\frac{1}{2}$ or $\frac{3}{4}$ inches long, made of moderately thick sheet brass; the jet may be of any desired length, and curved as convenience requires. The pipe is then to be attached to a minute box-hand-bellows, and the instrument is ready for use. Should any prefer it, a small foot-bellows, with flexible tube, may be used instead of the hand-bellows.

*Directions.*—Warm the cylinder to the desired temperature, over a small spirit lamp; gently force the air from the bellows into the tooth cavity. In passing through the cylinder, the air is heated. Care must be taken that the temperature is not raised too high.

When the cylinder is of proper thickness, say 18 or 20 of Stubb's gauge plate, and the outside properly polished, to prevent radiation, it retains its heat for several minutes. By coating it with silver and burnishing, it retains it still longer.

By the use of this instrument, a cavity, which has become flooded when partly filled, may be perfectly dried, and the operation finished, without the removal of the gold already inserted. Those who try the instrument, we think, will not be content without it. TAFT & WATT.

